# Supplementary material for: Evaluation of a package of risk-based pharmaceutical and lifestyle interventions in patients with hypertension and/or diabetes in rural China: A pragmatic cluster randomised controlled trial
Source: PLoS Med. 2021 Jul 1;18(7):e1003694. doi: 10.1371/journal.pmed.1003694 (PMC8284676; doi:10.1371/journal.pmed.1003694)
Supplement: S1 Text — Table A: Comparison of primary and secondary outcome disease event rates between the intervention and control arms (cluster-level data analysis). Table B: Comparison of systolic and diastolic blood pressure outcomes between the intervention and control arms (cluster-level data analysis). Table C: Comparison of time to first CVD event and time to CVD mortality outcomes between the intervention and control arms (cluster-level data analysis). Table D: Participant characteristics of per-protocol analysis. Table E: Comparison of event rates between the intervention and control groups in the per-protocol analysis. Table F: Comparison of time to first CVD event and time to CVD mortality outcomes between the intervention and control arms in the per-protocol analysis. Table G: Comparison of systolic and diastolic blood pressure outcomes between the intervention and control arms in the per-protocol analysis. Table H: Subgroup analysis for CVD event rates. Table I: Comparison of adverse events between the intervention and control arms. (DOCX) [file pmed.1003694.s002.docx]

**S1 Text**

**Contents**

1. Additional information on cluster-level statistical analyses methods
2. Table A. Comparison of primary and secondary outcome disease event rates between intervention and control arms (cluster-level data analysis).
3. Table B. Comparison of systolic and diastolic blood pressure outcomes between intervention and control arms (cluster-level data analysis).
4. Table C. Comparison of time to the first CVD event and time to CVD mortality outcomes between intervention and control arms (cluster-level data analysis).
5. Table D. Subject characteristics of per-protocol analysis.
6. Table E. Comparison of event rates between intervention and control groups in per-protocol analysis.
7. Table F. Comparison of time to the first CVD event and time to CVD mortality outcomes between intervention and control arms in per-protocol analysis.
8. Table G. Comparison of systolic and diastolic blood pressure outcomes between intervention and control arms in per-protocol analysis.
9. Table H. Subgroup analysis for CVD event rates.
10. Table I. Comparison of adverse events between intervention and control arms
11. References

**Additional information on cluster-level statistical analyses methods.**

A cluster-level analysis was conducted on top of the individual analysis. We summarized the event rates by cluster, and the overall median and range of event rates for each arm were reported. The cluster-level summary event rates were compared between two arms using an independent sample t-test. The mean and standard deviation (SD) of cluster-specific means of blood pressures and time to event outcomes were reported. Independent sample t-tests were used to compare the mean differences of cluster-specific mean changes in blood pressures between two arms. The cluster-level mean time to event outcomes was compared between two arms using an independent sample t-test.

**Table A. Comparison of primary and secondary outcome disease event rates between intervention and control arms (cluster-level data analysis).**

| **Outcome** | **Intervention (median & range event rate)^a^** | **Control (median & range event rate)^a^** | **Mean difference (95% CI)^b^** | **P-value** |
| --- | --- | --- | --- | --- |
| CVD event | 1.89 (0.50 to 3.28) | 1.97 (1.11 to 4.78) | -0.25 (-0.63 to 0.13) | 0.194 |
| CVD mortality | 0.43 (0.00 to 1.86) | 0.37 (0.00 to 1.13) | -0.02 (-0.18 to 0.14) | 0.790 |
| CHD event | 0.22 (0.00 to 1.86) | 0.29 (0.00 to 2.08) | -0.08 (-0.25 to 0.09) | 0.346 |
| CHD mortality | 0.08 (0.00 to 1.86) | 0.11 (0.00 to 0.57) | 0.01 (-0.12 to 0.14) | 0.879 |
| Stroke event | 1.58 (0.00 to 3.28) | 1.62 (0.00 to 4.05) | -0.17 (-0.54 to 0.20) | 0.351 |
| Stroke mortality | 0.26 (0.00 to 0.66) | 0.31 (0.00 to 0.92) | -0.04 (-0.15 to 0.06) | 0.413 |

CVD = cardiovascular disease. CHD = Coronary Heart Disease.

The total person years of follow-up were 39,657 and 43,557 for intervention group and control group respectively.

^a^ Cluster-level summary data are median (range) event rates (per 100 person-year) based on the separate cluster-specific event rates for each arm in a study duration of 3 years.

^b^ Mean difference between intervention and control cluster-level summary event rates, with 95% CI calculated using a t-test.

**Table B. Comparison of systolic and diastolic blood pressure outcomes between intervention and control arms (cluster-level data analysis).**

|  | **Intervention (mean [SD])^a^** | **Control (mean [SD])^a^** | **Mean difference (95% CI)^b^** | **P-value** |
| --- | --- | --- | --- | --- |
| **Systolic blood pressure (mmHg)** | | | | |
| Baseline | 135.8 (3.08) | 135.8 (3.55) |  |  |
| Endline | 131.8 (1.71) | 133.3 (1.74) | -1.41 (-2.73 to -0.09) | 0.037 |
| **Diastolic blood pressure (mmHg)** | | | | |
| Baseline | 81.6 (1.59) | 81.7 (1.72) |  |  |
| Endline | 79.3 (1.18) | 80.5 (1.04) | -1.05 (-1.77 to -0.34) | 0.005 |

^a^ Cluster-level summary data are mean (SD) of all the separate cluster-specific means.

^b^ Intervention minus control mean difference based on cluster-level summary means difference (i.e. individual's endline – baseline), with 95% confidence intervals calculated using a t-test.

**Table C. Comparison of time to the first CVD event and time to CVD mortality outcomes between intervention and control arms (cluster-level data analysis).**

|  | **Intervention (mean [SD])^a^** | **Control (mean [SD])^a^** | **Mean difference (95% CI)^b^** | **P-value** |
| --- | --- | --- | --- | --- |
| Time to the first CVD event | 544.4 (106.7) | 557.8 (66.7) | -13.5 (-57.1 to 30.1) | 0.539 |
| Time to the CVD mortality | 607.4 (158.9) | 616.7 (143.2) | -9.24 (-86.7 to 68.2) | 0.812 |

^a^ Cluster-level summary data are mean (SD) number of days of all the separate cluster-specific means but excluded censored outcomes.

^b^ Intervention minus control mean difference based on cluster-level summary mean time-to-first event values, with 95% confidence intervals calculated using a t-test

**Table D. Subject characteristics of per-protocol analysis.**

| **Patients’ characteristics** | **Intervention (n= 642)** | **Control (n=12,968)** | **Total** |
| --- | --- | --- | --- |
| Number of clusters | 32 | 33 | 65 |
| Mean age (years) | 65.1 (5.9) | 64.2 (6.1) | 64.3 (6.1) |
| Sex |  |  |  |
| Male | 315 (49.1%) | 6,426 (49.6%) | 6,741 (49.5%) |
| Female | 327 (50.9%) | 6,542 (50.4%) | 6,869 (50.5%) |
| Body mass index (kg/m^2^) | 24.6 (3.1) | 23.9 (2.9) | 24.0 (2.9) |
| Occupation |  |  |  |
| Farmer | 568 (89.6%) | 11,381 (90.2%) | 11,949 (90.2%)) |
| Worker | 10 (1.6%) | 163 (1.3%) | 173 (1.3%) |
| Technician | 2 (0.3%) | 52 (0.4%) | 54 (0.4%) |
| Administrative staff | 3 (0.5%) | 7 (0.1%) | 10 (0.1%) |
| Service worker | 2 (0.3%) | 49 (0.4%) | 51 (0.4%) |
| Personal business | 8 (1.3%) | 143 (1.1%) | 151 (1.1%) |
| Retired | 13 (2.1%) | 139 (1.1%) | 152 (1.1%) |
| Others | 28 (4.4%) | 679 (5.4%) | 707 (5.3%) |
| Educational level |  |  |  |
| Primary school and below | 493 (76.8%) | 9,763 (75.4%) | 10,256 (75.4%) |
| High school | 134 (20.9%) | 2,844 (22.0%) | 2,978 (21.9%) |
| College and above | 15 (2.3%) | 349 (2.7%) | 364 (2.7%) |
| Marital status |  |  |  |
| Married | 597 (94.2%) | 12,325 (95.8%) | 12,922 (95.7%) |
| Single, divorced or widowed | 37 (5.8%) | 543 (4.2%) | 580 (4.3%) |
| Mean annual per capita income (USD) | 1,486 (1,300) | 1,759 (2,773) | 1,745 (2,721) |
| Diagnosis |  |  |  |
| Hypertension with/without type 2 diabetes | 515 (80.2%) | 9,054 (69.8%) | 9,569 (70.3%) |
| Type 2 diabetes without hypertension | 127 (19.8%) | 3,914 (30.2%) | 4,041 (29.7%) |

Data are mean (SD) and n (%).

**Table E. Comparison of event rates between intervention and control arms in per-protocol analysis.**

| **Outcome** | **Intervention^a^** | | **Control^a^** | | **Crude IRR (95% CI)^b^** | **P-value** | **Adjusted IRR (95% CI)^c^** | **P-value** |
| --- | --- | --- | --- | --- | --- | --- | --- | --- |
|  | **N (%)** | **Event rate** | **N (%)** | **Event rate** |  |  |  |  |
| CVD event^d^ | 30 (4.67%) | 1.58 | 742 (5.72%) | 1.94 | 0.81 (0.55 to 1.20) | 0.298 | 0.74 (0.50 to 1.10) | 0.134 |
| CVD mortality | 9 (1.40%) | 0.47 | 168 (1.30%) | 0.44 | 1.06 (0.54 to 2.08) | 0.855 | 0.98 (0.49 to 1.92) | 0.943 |
| CHD event | 1 (0.16%) | 0.05 | 102 (0.79%) | 0.27 | 0.20 (0.03 to 1.43) | 0.110 | 0.18 (0.02 to 1.29) | 0.088 |
| CHD mortality | 0 (0.00%) | 0.00 | 50 (0.39%) | 0.13 | Undefined^e^ |  | Undefined^e^ |  |
| Stroke event | 27 (4.21%) | 1.42 | 627 (4.84%) | 1.64 | 0.87 (0.58 to 1.30) | 0.489 | 0.79 (0.53 to 1.19) | 0.262 |
| Stroke mortality | 9 (1.40%) | 0.47 | 120 (0.92%) | 0.31 | 1.49 (0.76 to 2.94) | 0.241 | 1.41 (0.71 to 2.78) | 0.324 |

Intervention n = 642, control n = 12,968. CVD = cardiovascular disease. HR = hazard ratio.

The total person years of follow-up were 1,904 and 38,340 for intervention group and control group respectively.

^a^ Individual-level summary statistics are number of events (%); event rate per 100 person- study duration, where % = total number of events / total number of individuals (per arm). Note for all “event” outcomes (as opposed to “mortality” outcomes) individuals may experience >1 event, and so the n (%) statistics are based on the occurrence/non-occurrence of the event per person only, but for event outcomes the rate statistics are based on the full count of events that occurred per person.

^b^ The intervention minus control crude IRRs were obtained via a Poisson mixed-effect model (primary outcome) or a Poisson or negative-binomial mixed-effect model (secondary outcomes: depending on the level of dispersion) with a single fixed effect of treatment arm and a random intercept for cluster. The 95% CIs and p-values were calculated based on the Wald-statistic. In all crude analyses there were no missing patients.

^c^ The intervention minus control adjusted IRRs were obtained via a Poisson mixed-effect model (primary outcome) or a Poisson or negative-binomial mixed-effect model (secondary outcomes: depending on the level of dispersion) with fixed effects for treatment arm, patient age, sex, body mass index, occupation (farmer, else), educational level (primary school and below, high school and above), marital status (married, else), income (less than US$1,644, US$1,644 and above) and diagnosis (hypertension without type 2 diabetes, type 2 diabetes without hypertension, hypertension with type 2 diabetes), and a random intercept for cluster. The 95% CIs and p-values were calculated based on the Wald-statistic. 12 (1.9%) and 463 (3.6%) patients in the intervention and control arms respectively were excluded in the adjusted analysis for each of the outcomes due to missing covariate data.

^d^ The intra-cluster correlation coefficient (ICC) for the CVD event was 0.012 which was determined by dividing the between-cluster variance by total variance. The variance terms were estimated by fitting an unconditional mixed effects model (Poisson / negative-binomial linked assumed depended on the dispersion level) with only a random intercept for clusters.^1,2^

^e^ Undefined: Estimation cannot be converged in the mixed effect models.

**Table F. Comparison of time to the first CVD event and time to CVD mortality outcomes between intervention and control arms in per-protocol analysis.**

|  | **Intervention (mean [SD])^a^** | **Control (mean [SD])^a^** | **Crude HR (95% CI)^b^** | **P-value** | **Adjusted HR (95% CI)^c^** | **P-value** |
| --- | --- | --- | --- | --- | --- | --- |
| Time to first CVD event | 690.2 (280.0) | 560.7 (315.1) | 0.77 (0.52 to 1.14) | 0.192 | 0.70 (0.47 to 1.04) | 0.079 |
| Time to CVD mortality | 593.3 (356.6) | 655.6 (297.1) | 1.07 (0.55 to 2.10) | 0.841 | 0.98 (0.50 to 1.94) | 0.952 |

Intervention n = 642, control n = 12,968. CVD = cardiovascular disease. HR = hazard ratio.

^a^ Individual-level summary data are presented as mean (SD) in number of days, but excluded censored outcomes.

^b^ The intervention minus control crude HRs were obtained using a mixed-effects Cox proportional hazard model with a single fixed effect of treatment arm and a random intercept for cluster. The 95% CIs and p-values were calculated based on the Wald-statistic. In both crude analyses there were no missing patients.

^c^ The intervention minus control adjusted HRs were obtained using a mixed-effects Cox proportional hazard model with fixed effects for treatment arm, patient age, sex, body mass index, occupation (farmer, else), educational level (primary school and below, high school and above), marital status (married, else), income (less than US$1,644, US$1,644 and above), diagnosis (hypertension without type 2 diabetes, type 2 diabetes without hypertension, hypertension with type 2 diabetes), and a random intercept for cluster. The 95% CI and p-values were calculated based on the Wald-statistics. 12 (1.9%) and 463 (3.6%) patients in the intervention and control arms respectively were excluded in the adjusted analysis for each of the outcomes due to missing covariate data.

**Table G. Comparison of systolic and diastolic blood pressure outcomes between intervention and control arms in per-protocol analysis.**

|  | **Intervention**  **(mean [SD])** | **Control (mean [SD])** | **Crude mean difference (95% CI)^a^** | **P-value** | **Adjusted mean difference (95% CI)^b^** | **P-value** |
| --- | --- | --- | --- | --- | --- | --- |
| **Systolic blood pressure (mmHg)** | | | | | | |
| Baseline | 136.9 (12.1) | 135.9 (11.4) |  |  |  |  |
| Endline | 133.3 (9.61) | 133.4 (9.71) | -0.64 (-1.88 to 0.60); | 0.315 | -1.04 (-2.24 to 0.16); | 0.089 |
| **Diastolic blood pressure (mmHg)** | | | | | | |
| Baseline | 81.7 (7.46) | 81.8 (6.64) |  |  |  |  |
| Endline | 79.6 (6.95) | 80.6 (6.30) | -1.25 (-2.02 to -0.49); | 0.001 | -1.32 (-2.05 to -0.59); | <0.001 |

Intervention n = 642, control n = 12,968.

^a^ The intervention minus control crude mean endline differences were obtained via a linear mixed-effects model with a single fixed effect of treatment arm and a random intercept for cluster. The 95% CI and p-values were calculated based on the t-statistic. In both crude analyses there were no missing patients.

^b^ The intervention minus control adjusted mean endline differences were obtained via a linear mixed-effects model with fixed effects for treatment arm, patient age, sex, body mass index, occupation (farmer, else), educational level (primary school and below, high school and above), marital status (married, else), income (less than US$1,644, US$1,644 and above), diagnosis (hypertension without type 2 diabetes, type 2 diabetes without hypertension, hypertension with type 2 diabetes), and a random intercept for clusters. The 95% CI and p-values were calculated based on the Wald-statistics. 56 (8.7%) and 1,586 (12.2%) patients in the intervention and control arms respectively were excluded in the adjusted analysis of systolic blood pressure, and 56 (8.7%) and 1,585 (12.2%) patients in the intervention and control arms respectively were excluded in the adjusted analysis of diastolic blood pressure due to missing covariate data or outcomes.

**Table H. Subgroup analysis for CVD event rates**

| **Subgroup** | **Intervention^a^** | | **Control^a^** | | **Crude IRR (95% CI)^b^** | **P-value** | **Adjusted IRR (95% CI)^c^** | **P-value** |
| --- | --- | --- | --- | --- | --- | --- | --- | --- |
|  | **N (%)** | **Event rate** | **N (%)** | **Event rate** |  |  |  |  |
| Patients with hypertension^d^ | 524 (5.95%) | 2.01 | 609 (6.60%) | 2.24 | 0.87 (0.73 to 1.03) | 0.096 | 0.86 (0.73 to 1.02) | 0.070 |
| Patients with diabetes only^d^ | 238 (5.19%) | 1.75 | 265 (4.80%) | 1.62 | 1.10 (0.79 to 1.54) | 0.575 | 1.09 (0.79 to 1.49) | 0.597 |
| Low income^e^ | 546 (5.99%) | 2.02 | 544 (6.40%) | 2.17 | 0.86 (0.70 to 1.05) | 0.145 | 0.87 (0.72 to 1.05) | 0.153 |
| High income^e^ | 216 (5.06%) | 1.71 | 330 (5.29%) | 1.79 | 0.94 (0.74 to 1.20) | 0.627 | 0.92 (0.72 to 1.16) | 0.467 |
| Primary school and below^f^ | 630 (6.08%) | 2.06 | 680 (6.14%) | 2.08 | 0.93 (0.76 to 1.13) | 0.469 | 0.90 (0.75 to 1.09) | 0.292 |
| High school and above^f^ | 131 (4.39%) | 1.48 | 192 (5.31%) | 1.79 | 0.78 (0.58 to 1.06) | 0.118 | 0.82 (0.60 to 1.12) | 0.206 |

^a^ Individual-level summary statistics are number of events (%); event rate per 100 person- study duration, where % = total number of events / total number of individuals (per arm).

^b^ IRR = incidence rate ratio. The intervention minus control crude IRRs were obtained via a Poisson or negative-binomial mixed-effect model (depending on the level of dispersion) with a single fixed effect of treatment arm and a random intercept for cluster. The 95% CIs and p-values were calculated based on the Wald-statistic. In all crude analyses there were no missing patients.

^c^ The intervention minus control adjusted IRRs were obtained via a Poisson or negative-binomial mixed-effect model (depending on the level of dispersion) with fixed effects for treatment arm, patient age, sex, body mass index, occupation (farmer, else), educational level (primary school and below, high school and above), marital status (married, else), income (less than US$1,644, US$1,644 and above) and diagnosis (hypertension without type 2 diabetes, type 2 diabetes without hypertension, hypertension with type 2 diabetes), and a random intercept for cluster. The 95% CI and p-values were calculated based on the Wald-statistic.

^d^ The total person years of follow-up were 25,044 and 27,230 for intervention group and control group respectively in patients with hypertension, while the total person years of follow-up were 13,613 and 16,327 for intervention group and control group respectively in patients with diabetes only. The intra-cluster correlation coefficients (ICC) for the CVD event was 0.012 and 0.060 respectively in patients with hypertension and diabetes only, which were determined by dividing the between-cluster variance by total variance. The variance terms were estimated by fitting an unconditional mixed effects model (Poisson / negative-binomial linked assumed depended on the dispersion level) with only a random intercept for clusters.^1,2^

^e^ The cutoff of low and high income is defined as US$1,644. The total person years of follow-up were 27,000 and 25,076 for intervention group and control group respectively in low-income patients, while the total person years of follow-up were 12,657 and 18,480 for intervention group and control group respectively in high-income patients. The intra-cluster correlation coefficients (ICC) for the CVD event was 0.025 and 0.022 respectively in low-income and high-income patients, which were determined by dividing the between-cluster variance by total variance.

^f^ The total person years of follow-up were 30,645 and 32,661 for intervention group and control group respectively in patients with low education level, while the total person years of follow-up were 8,870 and 10,725 for intervention group and control group respectively in patients with high education level. The intra-cluster correlation coefficients (ICC) for the CVD event was 0.025 and 0.033 respectively in patients with low and high education level, which were determined by dividing the between-cluster variance by total variance.

**Table I. Comparison of adverse events between intervention and control arms**

|  | **Intervention**  **N (%)** | **Control**  **N (%)** | **Crude OR (95% CI)^a^** | **P-value** | **Adjusted OR (95% CI)^b^** | **P-value** |
| --- | --- | --- | --- | --- | --- | --- |
| Total adverse event | 148 (1.1%) | 185 (1.3%) | 0.946 (0.531 to 1.686) | 0.850 | 0.927 (0.514 to 1.673) | 0.802 |
| Gastrointestinal discomfort | 57 (0.4%) | 77 (0.5%) | 1.017 (0.889 to 1.163) | 0.807 | 1.027 (0.891 to 1.185) | 0.709 |
| Dizziness | 30 (0.2%) | 43 (0.3%) | 1.012 (0.882 to 1.161) | 0.865 | 1.012 (0.875 to 1.170) | 0.871 |
| Hypodynamia | 17 (0.1%) | 33 (0.2%) | 1.017 (0.886 to 1.168) | 0.808 | 1.021 (0.882 to 1.182) | 0.778 |
| Undefined bleeding | 31 (0.2%) | 4 (<0.1%) | 0.964 (0.840 to 1.108) | 0.607 | 0.962 (0.831 to 1.115) | 0.608 |
| Other unclassified conditions | 13 (0.1%) | 28 (0.2%) | 1.017 (0.885 to 1.167) | 0.816 | 1.017 (0.878 to 1.177) | 0.826 |

Intervention n = 13,385, control n = 14,745.

^a^ The intervention minus control crude odds ratio (OR) was obtained via a generalised linear mixed-effects model with a single fixed effect of treatment arm and a random intercept for cluster. There were no missing patients in all crude analysis.

^b^ The intervention minus control adjusted OR was obtained via a generalised linear mixed-effects model with fixed effects for treatment arm, patient age, sex, body mass index, occupation (farmer, else), educational level (primary school and below, high school and above), marital status (married, else), income (less than US$1,644, US$1,644 and above), diagnosis (hypertension without type 2 diabetes, type 2 diabetes without hypertension, hypertension with type 2 diabetes), and a random intercept for clusters. In all adjusted analyses 1,153 (8.6%) and 1,470 (10.0%) patients in the intervention and control arms, respectively, were excluded due to missing covariate data.

**References:**

1. Nakagawa S, Schielzeth H. Repeatability for Gaussian and non‐Gaussian data: a practical guide for biologists. Biological Reviews. 2010 Nov;85(4):935-56.
2. Nakagawa S, Johnson PC, Schielzeth H. The coefficient of determination R 2 and intra-class correlation coefficient from generalized linear mixed-effects models revisited and expanded. Journal of the Royal Society Interface. 2017 Sep 30;14(134):20170213.
