# Supplementary material for: Evaluation of a package of risk-based pharmaceutical and lifestyle interventions in patients with hypertension and/or diabetes in rural China: A pragmatic cluster randomised controlled trial
Source: PLoS Med. 2021 Jul 1;18(7):e1003694. doi: 10.1371/journal.pmed.1003694 (PMC8284676; doi:10.1371/journal.pmed.1003694)
Supplement: Attachment — Submitted filename: wei.pdf [file pmed.1003694.s003.pdf]

This paper describes a trial of interventions for people with hypertension or diabetes in rural China.

I was asked for a statistical report and I interpret that to include all aspects of the design and conduct of the study.

Points of detail

Page 2 For me township means an urban area in South Africa reserved primarily for the black population during the time of apartheid. I believe it has a different specialist meaning in the US. I am not clear that township hospital is the best phrase. Later these are called rural primary care facilities which is not what I would understand by township hospital. I would suggest rural primary care facilities would be better throughout unless the authors can think of another phrase.

Page 15 I think strictly speaking these are risk ratios not rate ratios as time is not involved in the model.

Page 15 It is not clear to me why the authors do not consider event and mortality analyses as time to event outcomes.

Page 15 Why switch to odds ratios for the adherence and adverse event analyses?

Page 17 For the record not adjusting for multiplicity is fine.

Page 19 Income seems quite skewed so perhaps it would be better here and in Table 1 to report medians and quartiles. We could also use some context to tell us how much USD 1500 buys in rural China. I appreciate this is often difficult but the annual cost of the recommended package of medication which is finally reported on page 32 is helpful. We can then see it is of the order of USD 450 - 500 per year but page 32 seems a long way away from here. Could we have some more detail here?

Page 20 We now see rates per person year which is not, as I read it, what the statistical methods promised us for the primary analysis. This needs clarifying.

Supplementary table S4 There do seem to be differences between the control group and the residue of the intervention group. For instance the case-mix is different as the intervention arm have odds 1.75 times higher of having hypertension compared to the control group (95% confidence interval 1.44 to 2.14).

Point of more substance

The intention to treat analysis answers the question of the policy maker: if I implement this in my health service what effect will it have on the health of my population? It does not answer the patient's question: doctor, if I take these pills, stop smoking, cut down on salt, and drink less alcohol will it help me? Unfortunately the per protocol analysis presented here does not do that as it compares people who took the treatment in the intervention arm with the totality of people in the control arm. What is needed is a comparison of people who took the treatment in the intervention arm with those people in the control arm who would have taken the treatment if they had been offered it. This is usually called complier average causal effect (CACE).

Summary

Suggestions for clarification and a more important point about the analysis.

Michael Dewey
