# Supplementary material for: Evaluation of a package of risk-based pharmaceutical and lifestyle interventions in patients with hypertension and/or diabetes in rural China: A pragmatic cluster randomised controlled trial
Source: PLoS Med. 2021 Jul 1;18(7):e1003694. doi: 10.1371/journal.pmed.1003694 (PMC8284676; doi:10.1371/journal.pmed.1003694)
Supplement: Attachment — Submitted filename: PLOS Medicine responses to comments.docx [file pmed.1003694.s004.docx]

Dear Dr. Veitch and Dr. Misra,

Many thanks to the reviewers for their comments. We have provided a point to point response below, and also revised the manuscript with track changes. In addition to the responses to the reviewer’s comments we have also made several track changes edits to correct typos and improve phrasing. We appreciate your further comments. Please feel free to contact me.

Best regards,

Xiaolin and all researchers

PLOS Medicine comments

Requests from the editors:

*Thank you for including the CONSORT flowchart and checklist with the manuscript; we also recommend you include a statement in the Methods section that the CONSORT for cluster trials guidance has been used to help in reporting the study, and include a citation/reference to the guideline (<https://pubmed.ncbi.nlm.nih.gov/22951546/>)

*Please update the CONSORT checklist and reupload, ensuring that you use section and paragraph numbers to indicate where the checklist item information can be found, rather than page numbers (as papers are repaginated for publication).

Answer: Thanks very much for the comments. We have included the statement and inserted the reference in the manuscript. We also updated the CONSORT checklist using sections and paragraph numbers.

-----------------------------------------------------------

Academic editor advice/comments:

I think this is quite a well done trial and the findings and explanation for the null finding are quite plausible. The failure to make a substantive increase in medication rates, and short follow-up duration probably are the main drivers of the outcome.

For the former, they have argued it is mainly due to out of pocket costs for meds which I am sure is true but their process evaluation paper suggests there might be other factors at play such as level of trust with the township clinics.

It would have been nice to see some of the financial data on out of pocket costs and whether sub-groups based on income status shed any light but they state they are planning on including that in another paper.

I agree with the conclusion that system level interventions are needed, and notably primary health care in the Community Health Centres did not play a role in this intervention.

The findings contrast with studies like HOPE-4 which did provide free medication and got quite large BP reductions (but was not powered to look at CVD events). But I think in HOPE-4 there was also stronger community health worker engagement and this may also be key along with free meds. Our group found this also in Indonesia - <https://jamanetwork.com/journals/jamacardiology/fullarticle/2748764>

I am not that concerned about the statin issue except that despite including it as a focus in the intervention they did not measure lipid levels but one might assume with the medication rates achieved the LDL reduction would have been small.

Answer: Thanks very much for the positive comments. Indeed, we found that the primary care based intervention package were useful in improving a number of hypertension/ diabetes care, such as increased medicine update and reduced blood pressure. However, the health structure level issues, such as lack of insurance coverage for essential medicines and lack of trust to primary care providers, prohibited any success in improving medicine uptakes. Overall the baseline of drug-taking rate was very low: i.e., almost 0% on statin, less than 1/3 taking recommended anti-hypertensives. This trial also reflects it is hard to change prescribing behaviour unless there is structural change e.g., providing medicines for free plus community base health education, as reflected in HOPE 4 trial.

We have conducted the sub-analysis based on income (See in the Supplementary Table S8. Subgroup analysis for CVD event rates). There has been no significant differences shown between the intervention and control group based on different income groups. We also brought the costing issue up as a note in Table 1.

Our findings were compatible to the study in Indonesia in terms of improved drug uptake. We did not achieve the large reduction in blood pressure, which is likely because our baseline blood pressures were much lower (136/82 mmHg vs. 167/101 mmHg).

-----------------------------------------------------------

Comments from the reviewers:

Reviewer #1: See attachment (added below)

Michael Dewey

[see <https://www.editorialmanager.com/pmedicine/l.asp?i=612974&l=Q5JL4D1D>] (attached below)

This paper describes a trial of interventions for people with hypertension or diabetes in rural China.

I was asked for a statistical report and I interpret that to include all aspects of the design and conduct of the study.

**Points of detail**

Page 2 For me township means an urban area in South Africa reserved pri marily for the black population during the time of apartheid. I believe it has a diff t specialist meaning in the US. I am not clear that township hospital is the best phrase. Later these are called rural pri- mary care facilities which is not what I would understand by township hospital. I would suggest rural primary care facilities would be better throughout unless the authors can think of another phrase.

Answer: Thanks. We revised and clearly defined township hospitals as primary care facilities in line 168. We prefer to use township hospitals because township hospital is a term that is widely understood in our setting, and we used this term in our protocol and other related published manuscripts.

Page 15 I think strictly speaking these are risk ratios not rate ratios as time is not involved in the model.

Answer: We assume this sentence of this comment is referring to the text describing our GLMMs that used log-links and Poisson/negative binomial errors as producing incidence rate ratio results in relation to the effect of the intervention. Assuming this is correct then we can confirm that these are indeed incidence rate ratios as the outcome is a count variable (not a binary/binomial variable) and time is explicitly included in the model as an individual-level log-offset variable (a fixed-effect with a coefficient of 1). Hence, in such models the difference in incidence/event rates in each arm is computed on a log-scale in the model, which once exponentially transformed results in an incidence/event rate ratio as presented. We have therefore added an explanation that the relevant models included such an offset.

Page 15 It is not clear to me why the authors do not consider event and mortality analyses as time to event outcomes.

Answer: In relation to the second sentence of this comment, we believe that most public health researchers and more importantly certainly most policy makers and stakeholders are used to seeing incidence rates and can interpret them easily and “get a feel” for their magnitude compared to other diseases and contexts they are familiar with. Therefore, the natural effect measure of an incidence rate ratio makes sense from this perspective. However, survival analyses that typically result in hazard ratios are less easily interpretable and relatable to, particularly, policy makers and stakeholders. Therefore, we presented such results for our primary outcome should other researchers need them (e.g. to compare in meta-analyses), but we do not think it necessary to provide them instead of our incidence rate ratio analyses (or in addition to those analyses for the relevant secondary outcomes). We therefore do not believe this requires any changes.

Page 15 Why switch to odds ratios for the adherence and adverse event analyses?

Answer: This was planned because these outcomes were seen as binary at the individual level either by definition (i.e. adhered/not adhered) or in practice (adverse events may occur more than once per individual, but we did not find that in our data). Therefore, as the typical analysis approach for binary outcomes is via a logistic regression we followed this approach, which results in odds ratios once the model coefficients are back-transformed for interpretability.

Page 17 For the record not adjusting for multiplicity is fine.

Answer: Thank you for sharing your view.

Page 19 Income seems quite skewed so perhaps it would be better here and in Table 1 to report medians and quartiles. We could also use some context to tell us how much USD 1500 buys in rural China. I appreciate this is often difficult but the annual cost of the recommended package of medication which is fi reported on page 32 is helpful. We can then see it is of the order of USD 450 - 500 per year but page 32 seems a long way away from here. Could we have some more detail here?

Answer: We have revised them to medians and 25% and 75% quartiles in Table 1 and added the cost of the full package of intervention medicines in the legend as a reference of purchasing capacity in local setting.

Page 20 We now see rates per person year which is not, as I read it, what the statistical methods promised us for the primary analysis. This needs clarifying.

Answer: We are not clear exactly what part of the methods the reviewer believes is being contradicted by the way the results are presented for the primary outcome on page 20, but we do not see any inconsistency. To clarify, in our methods we stated that for the primary outcome we used Poisson/negative binomial GLMMs to estimate the treatment effect on the primary outcome “as an incidence rate ratio [IRR]”, which is indeed how the treatment effect is presented on page 20: “the crude IRR was 0.90 (95% CI: 0.74, 1.08; P = 0.259; Table 2).” We did also present the event rates per treatment arm prior to the treatment effect estimate presented in the previous sentence, which is maybe what has caused the reviewers comment given they mention “rates per person year”. However, this is simply the summary measure of the outcome within each arm to allow readers to understand the actual absolute value of the rates being compared in the rate ratio, which is a dimensionless metric and therefore hard to fully interpret without such additional information. Also, although these within-arm rates were not explicitly promised in the methods as per CONSORT guidelines it is recommended to present, as standard, the natural summary measure within all arms for all outcomes. Lastly, to clarify further if necessary: the treatment effect estimate (i.e. the IRR) is not affected by the choice of the denominator for the within-arm rates (e.g. per 100 person-years etc).

Supplementary table S4 There do seem to be diff	between the control group and the residue of the intervention group. For instance the case-mix is diff t as the intervention arm have odds 1.75 times higher of having hypertension compared to the control group (95% con- fi	interval 1.44 to 2.14).

Answer: We appreciate there is some evidence of imbalance between the per-protocol intervention and control groups, and this clearly represents a likely source of bias. However, we did adjust for various key characteristics, including diagnosis, that whether imbalanced or balanced were thought to be likely influence on the outcome and therefore should be adjusted for to minimise the effects of any imbalances following the creation of the PP groups. The adjustment variables are clearly stated in Table S5.

**Point of more substance**

The intention to treat analysis answers the question of the policy maker: if I implement this in my health service what effect will it have on the health of my population? It does not answer the patient’s question: doctor, if I take these pills, stop smoking, cut down on salt, and drink less alcohol will it help me? Unfortunately the per protocol analysis presented here does not do that as it compares people who took the treatment in the intervention arm with the totality of people in the control arm. What is needed is a comparison of people who took the treatment in the intervention arm with those people in the control arm who would have taken the treatment if they had been offered it. This is usually called complier average causal effect (CACE).

Answer: We appreciate the insightful comment and accept the limitations and bias of the PP analysis, which was our protocol planned approach (although not fully defined there). However, we are not in a position to attempt a CACE analysis and we do not believe it would add to the study given the extremely low level of adherence/compliance in our intervention arm, which clearly prohibits any robust estimation of the actual treatment effect/treatment efficacy via any methods, as opposed to the effect of treatment provision as robustly estimated by our ITT analyses. Clearly this problem equally applies to our PP analyses though and so we have added further discussion and acknowledgement of this severe limitation, concluding that such efficacy evidence would obviously simply require further study. See the first paragraph of the Discussion section for the additions.

**Summary**

Suggestions for clarification and a more important point about the analysis. Michael Dewey
-----------------------------------------------------------

Reviewer #2:

This is a very interesting study in the important area of improving primary prevention outcomes in low-resource settings. However, the results are disappointing and it seems that lack of efficacy is mainly driven by very low adherence. Even though the adherence issue takes up most of the discussion, I think that is not enough.
First, it is not clear why adherence was assessed only by self-report, and no validated tools were used.

Answer: Thanks for the comments. We discussed this during the designing stage but decided not to include other tools to collect adherence based on the following considerations:

1) Using electronic medical boxes would be the best way to record drug taking. However, at the time of trial (2015) functional and affordable electronic medical boxes were not available. Some reported technical challenges such as connectivity and battery lifetime, and they were expensive (>300 RMB/ 50 USD per box). And the size of electronic medical boxes may vary because patients were taking different medications in different doses. 2) We would not be able to persuade policy makers purchase the electronic medical boxes in a large scale after the trial given their constraints even in covering medication cost. 3) We did not include much longer questions than self reported adherence to reduce any disturbance of medical consultation for the reason that this is a pragmatic trial. That is, extra time of questionnaire data collection at each consultation will prolong the time and delay other patients who are in the waiting room. 4) We did not include the self-report adherence scales (such as the Morison Scale) because they did not measure the real drug-take-up rate.

Second, since adherence turned out to be a crucial factor, I would suggest expanding the analysis of the reasons for non-adherence. For example, you could consider adding a logistic regression of factors predicting lack of adherence.

Answer: Thanks. We will consider this in another paper because this paper aimed to report the main results of the trial as set in our analysis plan.

Aside from the adherence problem, it is not clear to me why aspirin was included in the standard drug package for patients without atherosclerotic CVD.

Answer: We included aspirin in the package because of the initial idea of polypill and later in most polypill trial (including the recent Lancet PolyIran trial which contained aspirin 81 mg). This is predicted to reduce ischemic CVD including ischemic stroke and coronary heart disease. More evidence only became available recently (for example, the ARRIVE trial by Gaziano, 2018) that low dose aspirin did not reduce CVD events among people who had moderate risk of CVD (10-20% in 10 years). However, our group of patients had high risk of CVD (>=20%)

Finally, the text will greatly benefit from being checked by a native English speaker. It contains several mistakes in the choice of words, for example "What was this study done?"

Answer: Thanks. It should be “Why was this study done”. We corrected it and also had native English speakers reviewed the manuscript and made necessary changes.

-----------------------------------------------------------

Reviewer #3:

The authors are reporting the results of a pragmatic trial in patients with hypertension and 10 year CVD risk of 20% or higher, and patients with diabetes regardless of their 10 year CVD risk. In this cluster design study, the intervention arm received a standardised package of medicines, individual advice on lifestyle change and adherence support. The control arm received the usual care. At 36 months, there was non-significant difference in CVD incidence rates.


1-      All severe CVD events were collected through Zhejiang CDC's surveillance system: can you describe accuracy, reliability and representativeness? Are 100 % of events recorded in this system?

Answer: We reported the Zhejiang CDC’s surveillance system elsewhere. It was referred in Line 262 as reference 25.

Gong W, Wei X, Liang Y, Zou G, Hu R, Deng S, et al. Urban and Rural Differences of Acute Cardiovascular Disease Events: A Study from the Population-Based Real-Time Surveillance System in Zhejiang, China in 2012. PloS one. 2016;11(11):e0165647.

We described the acute event registration, collection and validation, and its quality control. Collection was complete as it covered all hospitals in Zhejiang province, and also connected with the death registration system in Zhejiang. There are a number of validation process implemented to ensure its completeness and accuracy:

In the manuscript, we briefly presented the validation process (Line 257-261). We also referred to our published paper, which shows the following procedure of validation:

A strict registration and validation procedure was undertaken once an acute CVD event was detected. First, the public health department of the health care facility reviewed CVD reports submitted by clinical doctors before registering them in the system. Second, all reported new acute CVD events were reviewed and verified by staff in the CDCs from the county to the provincial level. Only when a reported event met the standards, would the CDC staff verify, register and refer it to the next level. After an acute CVD event was registered at the county level, the primary care facility, whose catchment area covers the case, was responsible to validate the event through home visits. Regular training on ICDSS operational guidelines was provided to health care workers for supervision of acute CVD events reported in their facilities.

2-      Line 258 : "in addition, family doctors asked participants if they had experienced any stroke or heart attack events that were diagnosed by hospitals at the 12th,….. This was then verified by the Validation Committee and included in the 261 records, if not previously"

Can the authors define how many CV events were revealed by this method and not originally detected by the surveillance system.
If this was high that means other events from patients who were not seen by participating doctors for follow up may be have been missed and that would lead to a major bias in the ITT analysis.

Answer: Only 9 of all 1,636 (0.55%) reported CVD events in this trial were revealed by follow-up of family doctors and others were recorded in the CVD surveillance system, which reflects that the CVD surveillance system have collected most of CVD events. We have added such information in line 424: “The majority (99.4%) of CVD events were collected through Zhejiang’s CVD surveillance system, while four and five CVD events from the intervention and control arms respectively, were collected via family doctors at their consultations.”

3-      Define cvd event in the paragraph starting with line 249  ,similar to how CHD and stroke were defined

Answer: All CVD events were defined according to the WHO MONICA definitions that was made compatible to ICD 10 codes. This included two categories: coronary heart disease and stroke. Detailed diagnoses of CHD and stroke under ICD 10 codes were presented in the following lines (now line 252-255).

4-      Is it possible to describe those who declined inclusion in the trial to make sure there is no systemic bias?

Answer: Figure 1 reported that 3,196 of 31,326 (10.2%) eligible patients declined to participate in the trial, i.e. refused to sign the consent form, thus the research team were not allowed to obtain their characteristic data based on our ethical approval. We can still get a clue from Table 1 regarding the bias issue. This illustrated that, at least in regard to the key characteristics of participants who were included, they were comparable between the intervention and control groups. Given the size of our trial we think it is reasonable to say that we had a low risk of bias due to randomised imbalances in important variables. We added this as one limitation of the study.

5-      Understand the reason for low adherence

Answer: Thanks. Many of the reasons, such as high cost of medicines, low insurance coverage, lack of low cost medicines, and lack of trust towards township hospital doctors were illustrated in our process evaluation.

6-      In this statement: clusters and participants were successfully followed up based on electronic medical records and/or CVD events reported via family doctors: how were medical records reviewed? what percentage is electronic?

Answer: Please see answers to question 2: 99.45% were electronic data from CVD surveillance system. For 0.55% of reported CVD patients, their paper-based medical record was reviewed by the Validation Committee and reported by the township hospital family doctors.

7-      English and grammar need to be improved in some areas for example in the method section: line 32:
We randomised 67 township hospitals to intervention (34) or control (33) arms, and 33 recruited 31,326 participants, (15,380 in the intervention arm and 15,946 in the control 34 arm), with no known CVD, and with hypertension and a 10-year CVD risk of 20% or 35 higher, and all patients with type 2 diabetes.

Although clear in the title, the diabetes inclusion criteria was a bit confusing here and  not clear until reading table 2. An easier way to read it would be participants with diabetes, or hypertension and …/ or could say participants with hypertension and 10 -year CVD risk…. And type 2 diabetes regardless of 10-year CVD risk…

Answer: Thanks. We revised this.

We used a pragmatic, parallel group, two-arm, controlled, superiority, cluster trial design. We randomised 67 township hospitals to intervention (34) or control (33) arms. A total of 31,326 participants were recruited with 15,380 in the intervention arm and 15,946 in the control arm. Participants had no known CVD. They were patients either with hypertension and a 10-year CVD risk of 20% or higher, or patients with type 2 diabetes regardless of their CVD risk.

Other examples:
line 604, shows incomplete sentence:
Given the interventions were shown to be feasible for family doctors [15] and patients [17].

Line 260: incomplete sentence
This was then verified by the Validation Committee and included in the records, if not previously.

Thanks, we revised them. We also have had two English speakers revised the manuscript.

8-      What were the adverse events?

Answer: We added the type of adverse events info in line 555-557 as below:

Of 333 reported adverse events, 134 (40.2%) were gastrointestinal discomfort, 73 (21.9%) were dizziness, 50 (15.0%) were hypodynamia, 35 (10.5%) were undefined bleeding, and 41 (12.4%) were other unclassified conditions.

9-      10 year CVD risk :
-       What is the 10 year CVD risk in each arm in those with hypertension?

Answer: The median 10-year CVD risk of patients with hypertension was 26.5% in the intervention arm and 26.3% in control arm. We have added this info in Table 1. We presented the median value because this value is highly skewed towards the lower risk end.

-       Although patients with diabetes are at higher risk for CVD compared to those without diabetes, the risk of CVD is not the same in all patients. For example,  the risk of CVD in diabetes may be higher in patients  depending on presence of  microalbuminuria, smoking, etc… so having an imbalance of those contributing factors between groups,  may also lead to imbalance of  CVD risk as well and therefore bias the results.

Answer: As this was a large randomised controlled trial in expectation there should be no imbalance in terms of any characteristic or any combination of characteristics between the intervention and control groups, and any risk of imbalance gets smaller as the trial gets bigger (and we randomised ~30k individuals). Therefore, internal validity is not affected in any way by how patient diagnoses are defined or determined or by how patients are excluded/included in the trial etc as long as that is all done prior to randomisation, as it was. Although the lack of an imbalance and any threat to internal validity can never be fully verified empirically the data we collected on key characteristics, including diagnosis, showed that we appeared to have very little imbalance in the key characteristics we measured between arms. See Table 1.

10-     Line 609, would need to describe in the methods how patients were informed about their CVD risk

Answer: Patient were verbally informed that they had over 20% of having CVD in the next ten years, and they need to adopt activities in reducing their CVD risk, such as treating hypertension or diabetes and making healthy lifestyle changes.

11-     Line  635, although it makes sense, we do not have clear understanding based on this study

Answer: We revised this as:

Our study, along with other studies from HICs, showed that interventions that reduce CVD risks at the population level would be unlikely to have an impact unless structural health system factors such as the availability of essential medicines are addressed first.
