# Supplementary material for: Evaluation of a package of risk-based pharmaceutical and lifestyle interventions in patients with hypertension and/or diabetes in rural China: A pragmatic cluster randomised controlled trial
Source: PLoS Med. 2021 Jul 1;18(7):e1003694. doi: 10.1371/journal.pmed.1003694 (PMC8284676; doi:10.1371/journal.pmed.1003694)
Supplement: Attachment — Submitted filename: Response to comments June 2021.docx [file pmed.1003694.s005.docx]

Response to comments:

Re: Effect of a package of risk-based pharmaceutical and lifestyle interventions on patients with hypertension and/or diabetes in rural China: a pragmatic cluster randomised controlled trial" (PMEDICINE-D-20-05015R2)

Dear Dr. Turner,

Thanks very much for the effort of reviewing the manuscript. We would like to fully address the comments. Please see our responses below:

For Editor’s Requests:

We have fully addressed to each of the comment and made related changes in the manuscript using track changes. But we did not find the word “farmers” in Line 403 (from the submitted file).

For Reviewer 2:

We added a limitation:

Seventh, recalling and reporting bias may exist because we collected self-reported adherence to medicines. Electronic pill boxes were not available during our study, and even available, they may alter provider and patient’s behaviour.

Regarding the breakdowns of bleeding, we provide an extra supplementary table (Table S9) to include the breakdowns of all adverse events by the intervention and control arm, and added the sentence “No significant difference was identified in each of the adverse event (Table S9).”
